# Two New Cholic Acid Derivatives from the Marine Ascidian-Associated Bacterium *Hasllibacter halocynthiae*

**DOI:** 10.3390/molecules171012357

**Published:** 2012-10-22

**Authors:** Sung Hun Kim, Yun Kyung Shin, Young Chang Sohn, Hak Cheol Kwon

**Affiliations:** 1 Natural Medicine Center, Korea Institute of Science and Technology (KIST), Gangneung 210-340, Korea; Email: marineamigo@hotmail.com; 2 Division of Applied Marine Biotechnology and Engineering, Faculty of Marine Bioscience and Technology, Gangneung-Wonju National University, Gangneung 210-702, Korea; Email: ykshin@nfrdi.go.kr; 3 Aquaculture Management Division, National Fisheries Research and Development Institute, Busan 619-705, Korea; Email: ykshin@nfrdi.go.kr

**Keywords:** *Hasllibacter halocynthiae*, cholic acids, cholanic acid ketal, marine ascidian

## Abstract

The investigation of secondary metabolites in liquid cultures of a recently discovered marine bacterium, *Hasllibacter halocynthiae* strain KME 002^T^, led to the isolation of two new cholic acid derivatives. The structures of these compounds were determined to be 3,3,12-trihydroxy-7-ketocholanic acid (**1**) and 3,3,12-trihydroxy-7-deoxycholanic acid (**2**) through HRFABMS and NMR data analyses.

## 1. Introduction

Ascidians are marine invertebrates in the phylum Chordata. Among these abundant group members, *Halocynthia roretzi *is an edible ascidian with rock and artificial structures in shallow ocean waters as its habitats. Ascidians efficiently take up food particles and microorganisms by filter feeding seawater through a pair of siphons. This filter-feeding ability could make ascidians as an ideal source for the discovery of unique marine microorganisms. Although several bacteria associated with *Halocynthia roretzi* have been reported [1,2,3,4,5,6,7], the chemical-ecological interactions between the ascidian hosts and their associated bacteria is unclear. 

In our previous study, the bacterial strain KME 002^T^, isolated from the marine ascidian (*H. roretzi*) was identified as a novel species, *Hasllibacter halocynthiae*. This belongs to the *Roseobacter* clade. The members of this clade, which are ubiquitous in various marine habitats, produce a variety of bioactive compounds [8,9,10,11]. Three bile acid derivatives: 3,12-dihydroxy-7-ketocholanic acid, 12-hydroxy-3-ketoglycocholanic acid and nutriacholic acid, were identified as major secondary metabolites produced by *Hasllibacter halocynthiae* [5]. Bile acids are not only physiologically important agents for the digestion of dietary fats, carotenoids and vitamins [12], but also important therapeutic agents for treating cholestasis, gallstone, fatty liver, cardiovascular disease, obesity and diabetes in humans [13]. Bile acids are generally produced in eukaryotic cells and less than 15 bacterial strains are known to produce these metabolites [14,15,16,17]. 

**Figure 1:**
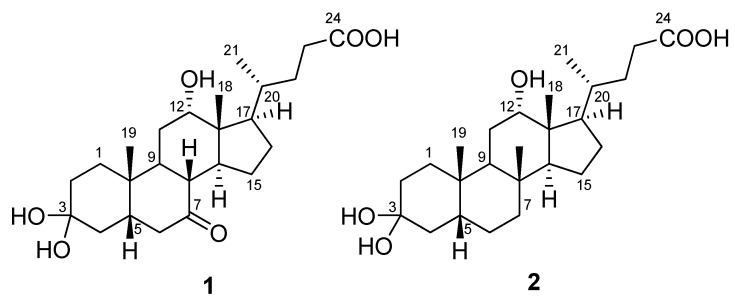
Structures of compounds **1** and **2**.

In our further investigations, unique bile acid derivatives produced by strain KME 002^T^ were observed. Two novel cholic acid derivatives, 3,3,12-trihydroxy-7-ketocholanic acid (**1**) and 3,3,12-trihydroxy-7-deoxycholanic acid (**2**), were isolated and identified from organic extracts of *H. halocynthiae *KME 002^T^. The structures of these compounds were determined by 2D-NMR and HRFABMS (high-resolution fast atom bombardment mass spectrometry). This paper describes the isolation and structural elucidation of these two novel cholic acid derivatives, compounds **1** and **2** (Figure 1). 

## 2. Results and Discussion

The sequence analysis of the 16S rRNA gene placed strain KME 002^T^ within the *Roseobacter *clade in Alphaproteobacteria, and the 16S rRNA gene sequence of strain KME 002^T^ showed similarities of 95.0, 94.7 and 94.5% to *Dinoroseobacter shibae*, *Roseovarius crassostreae* and *Pseudoruegeria aquimaris*, respectively. In addition, comprehensive analyses showed that KME 002^T^ had distinct phenotypic characteristics compared with its close phylogenetic relatives, suggesting a novel genus and species of the family *Rhodobacteraceae*, which was named *Hasllibacter halocynthiae*. Cultures of KME 002^T^ were deposited at the Japan Collection of Microorganisms (JCM 16214^T^) and the Korean Culture Center of Microorganisms (KCCM 90082^T^).

Strain KME 002^T^ was isolated from the siphon tissue of a marine ascidian, *Halocynthia roretzi*. To investigate secondary metabolite production by KME 002^T^, the strain was cultured in 32 one liter Erlenmeyer flasks, each containing 500 mL of A1 + C liquid medium (total 12 L) [10 g of starch (Difco), 2 g of yeast extract (Difco), 1 g of calcium carbonate (Aldrich, 98%) and 4 g of peptone (Difco) in 1 L sterilized distilled water]. Secondary metabolite production in the culture broth was monitored daily by HPLC analysis using an Agilent 1200 series LC system coupled with an Agilent 6120 Quadrupole mass spectrometer using a Phenomenex Luna C18(2) column (150 × 4.60 mm, 5 μm) eluted with a gradient of 10%–100% acetonitrile in water containing 0.05% formic acid for 30 min at a flow rate of 0.7 mL/min. Two major peaks were observed with retention times of 18.3 and 23.5 min on the fifth day of culture. At the end of the culture period (7 days), the culture broth of strain KME 002^T^ was extracted twice with ethyl acetate (EtOAc). The crude EtOAc extract was subjected to an initial separation using reverse-phase flash column chromatography, and the 60% and 80% aqueous acetonitrile fractions were purified with preparative HPLC (C18 column, 10 × 250 mm) to yield compounds **1** and **2**.

**Figure 2:**
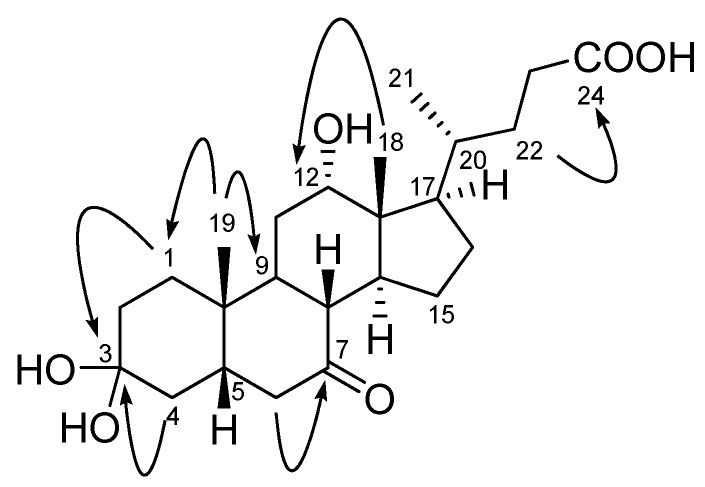
Key HMBC correlations of compound **1**.

Compound **1** was isolated as a colorless oil. Its molecular formula was deduced as C_24_H_38_O_6_ based on HRFABMS (obsd., [M–H_2_O+Na]^+^ at *m/z *427.2451) and ^13^C-NMR data. The IR spectrum of **1** displayed absorption bands at 3,479 and 1,714 cm^−1^, indicating the presence of hydroxyl and carbonyl functionalities. The ^1^H- and ^13^C-NMR data of **1** (Table 1) were almost identical to those of 3α,12α-dihydroxy-7-ketocholanic acid isolated from the culture broth of a sponge-associated *Psychrobacter* sp. [17]. However, there was no H-3 carbinol proton signal in the ^1^H-NMR spectrum of **1**, and the C-3 carbon signal was observed much further downfield at 101.7 ppm in the ^13^C-NMR spectrum of **1**. The C-3 quaternary carbon signal showed HMBC correlation with H_2_-2 and H_2_-4, indicating that a dihydroxyl group was attached at the C-3 position (Figure 2). The optical rotation value of **1** (+19.0 *c* 0.1, EtOH) was similar to that of compound **2** and 12α-hydroxy-7-deoxy-3-ketocholanic acid (+44.1 *c* 1.6, chloroform) [18], indicating that the absolute configurations of the chiral centers in **1** are identical to those of these two compounds. Thus, the structure of **1** was assigned as 3,3,12-trihydroxy-7-ketocholanic acid.

Compound **2** was obtained as colorless oil that was determined to be C_24_H_40_O_5_ based on HRFABMS ([M–H_2_O+Na]^+^ at *m/z* 413.2662) and NMR data (Table 1). The IR spectrum of **2** showed absorption bands almost identical to those of **1**. The ^1^H- and ^13^C-NMR spectra of compound **2** were similar to those of 3,12-dihydroxy-7-deoxycholanic acid [19], the major differences being the absence of an H-3 carbinol proton signal in the ^1^H-NMR spectrum of **2** and the downfield shift of the C-3 signal to 102.5 ppm in the ^13^C-NMR spectrum of **2**, as observed in **1**. The quaternary carbon signal at 102.5 ppm showed HMBC correlations with H_2_-2 and H_2_-4, indicating that a dihydroxyl group was attached to the C-3 carbon, again similar to the structure of **1**. In addition, the optical rotation values of **2** and 12α-hydroxy-7-deoxy-3-ketocholanic acid [18] were +25.0 (*c* 0.1, EtOH) and +44.1 (*c* 1.6, chloroform), respectively. These data indicated that the structure of **2** has the same absolute configuration as 3-dimethoxy-12-hydroxycholanic acid and 12α-hydroxy-7-deoxy-3-ketocholanic acid. Thus, the structure of **2** was determined to be 3,3,12-trihydroxy-7-deoxycholanic acid.

**Table 1:** ^1^H and ^13^C-NMR spectral data for compounds **1** and **2 **in methanol-*d*_4_.

Position	1			2		
	*δ*_H_ mult (*J*, Hz) *^a^*	*δ*_C_ *^b^*		*δ*_H_ mult (*J*, Hz) *^a^*	*δ*_C_ *^b^*	
1	1.67 m, 1.33 m	33.2	CH_2_	1.62 m, 1.15 m	34.2	CH_2_
2	1.75 m, 1.38 m	28.0	CH_2_	1.88 m, 1.25 m	28.1	CH_2_
3		101.7	C		102.5	C
4	1.72 m, 1.26 m	35.8	CH_2_	1.83, 1.57 m	34.6	CH_2_
5	2.02 m	45.9	CH	1.50 m	41.3	CH
6	2.97 dd (12.5, 6.0), 1.84 m	46.1	CH_2_	1.72 m, 1.48 m	28.4	CH_2_
7		215.1	C	1.86 m, 1.29 m	28.8	CH_2_
8	2.56 dd (11.5, 11.5)	50.9	CH	1.47 m	37.4	CH
9	2.23 m	37.4	CH	1.87 m	34.4	CH
10		36.2	C		35.6	C
11	1.77 m, 1.54 m	30.9	CH_2_	1.53 m	30.2	CH_2_
12	3.98 br t (3.0)	73.0	CH	3.96 br t (3.0)	74.2	CH
13		47.7	C		47.7	C
14	1.98 m	42.1	CH	1.60 m	49.5	CH
15	2.15 m, 1.01 m	25.5	CH_2_	1.62 m, 1.10 m	25.0	CH_2_
16	1.90 m, 1.29 m	28.9	CH_2_	1.44 m, 1.13 m	27.5	CH_2_
17	1.80 m	47.3	CH	1.84 m	48.3	CH
18	0.72 s	13.4	CH_3_	0.71s	13.3	CH_3_
19	1.23 s	23.4	CH_3_	0.94 s	23.8	CH_3_
20	1.41 m	36.7	CH	1.43 m	36.9	CH
21	1.01 d (6.5)	17.8	CH_3_	1.01 d (6.5)	17.7	CH_3_
22	1.79 m, 1.36 m	32.4	CH_2_	1.79 m, 1.33 m	32.5	CH_2_
23	2.35 ddd (15.0, 9.5, 5.0)	32.0	CH_2_	2.35 ddd (15.5, 10.0, 5.5)	32.2	CH_2_
	2.26 m			2.22 ddd (7.0, 9.5, 16.0)		
24		176.6	C		178.3	C

*^a^* 500 MHz; *^b^* 125 MHz; Reference chemical shifts: *δ*_H_ 3.31, *δ*_C_ 49.0 for methanol-*d*_4_.

**Figure 3:**
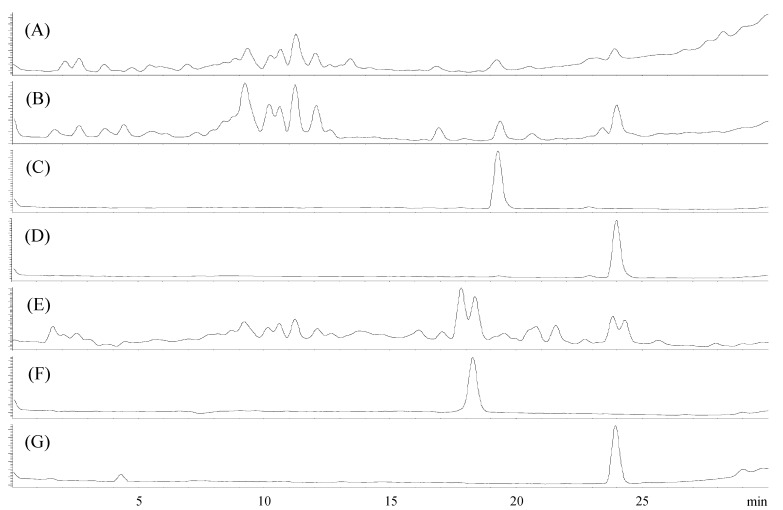
LC/MS chromatograms: (**A**) A1+C liquid medium, (**B**) Bacto peptone (Difco 211677) solution, (**C**) Cholic acid (Sigma C1129), (**D**) Deoxycholic acid (Sigma D2510), (**E**) Culture extract of strain KME 002^T^ (Day 7), (**F**) Compound **1**, (**G**) Compound **2**.

Compounds **1** and **2** may be produced by microbial transformation of cholic acid or deoxycholic acid in the liquid medium (Figure 3). Actually, cholic acid is often found in Bacto peptone which is an ingredient of the A1+C liquid medium [20]. However, the isolation of **1** and **2** is considered to contribute to a better understanding of the chemical diversity of steroidal structures because the 3,3-dihydroxylated steroid derivative is very rare in Nature. 

## 3. Experimental Procedures

### 3.1. General

Optical rotation was measured with a Perkin-Elmer model 343 polarimeter (Perkin-Elmer, Waltham, MA, USA). FT-IR spectra were acquired with a Nicolet iS10 spectrometer (Thermo Fisher Scientific, Waltham, MA, USA). ^1^H-, ^13^C-NMR and 2D NMR (gHSQC, gHMBC and gCOSY) data were obtained in CD_3_OD (*δ*_H_ 3.31 and *δ*_C_ 49.0) on a Varian Unity Plus 500 MHz NMR System (Varian, Palo Alto, CA, USA). Low-resolution mass spectra were recorded with an Agilent 1200 series LC system coupled with an Agilent 6120 Quadrupole mass spectrometer (Agilent Technologies, Santa Clara, CA, USA). HRFABMS data were obtained on a JEOL/JMS-AX505WA instrument at the National Center for Inter-University Research Facilities of Seoul National University. Cosmosil 75 C18-Prep (Nacalai Tesque, Kyoto, Japan) was used for reverse-phase flash column chromatography. Preparative HPLC separations were performed using a Gilson 321 HPLC system (Gilson, Middleton, WI, USA) equipped with a Phenomenex Luna C18(2) column (250 × 10.00 mm, 10 μm) and a differential refractive index detector (Shodex, RI-101). A YL9100 HPLC system (Younglin, Korea) equipped with a Phenomenex Luna C18(2) column (150 × 4.60 mm, 5 μm) and an ELSD ZAM 3000 detector (Schambeck SFD GmbH, Bad Honnef, Germany) was used for HPLC analysis. 

### 3.2. Collection, Isolation and Identification of Strain KME 002^T^

*Hasllibacter halocynthiae *sp. KME 002^T^ was isolated from a marine ascidian, *Halocynthia roretzi*, collected at a depth of 15 m (water temperature: 17 °C) near Kyung-Po beach in Korea (June 2007). The ascidian was collected and immediately washed with autoclaved seawater, and its atrial and branchial siphons were finely ground and diluted with filtered, autoclaved seawater at a ratio of 1:10. The diluted suspension (100 µL) was spread on an A1+C agar plate. The A1+C agar medium consists of 10 g starch (Difco Laboratories, Detroit, MI, USA), 4 g peptone (Difco), 2 g yeast extract (Difco), 1 g calcium carbonate (Aldrich, 98%) and 18 g agar (Difco) in 1 liter of filtered seawater. The plate was incubated for 3 weeks at 25 °C under aerobic conditions. KME 002^T^ was isolated as circular, convex and light red colonies on an A1+C agar plate. The isolate was stored at −80 °C in A1+C liquid medium supplemented with 20% glycerol (v/v). KME 002^T^ was identified using a polyphasic approach, including phenotypic, chemotaxonomic and genetic analyses [5]. Based on these polyphasic characteristics, strain KME 002^T^ was designated *Hasllibacter halocynthiae*, a novel genus and species of the family *Rhodobacteraceae*. KME 002^T^ was deposited at the Japan Collection of Microorganisms (= JCM 16214^T^) and the Korean Culture Center of Microorganisms (=KCCM 90082^T^).

### 3.3. Cultivation and Extraction

KME 002^T^ was cultured aerobically in A1+C liquid medium (25 mL) with shaking at 200 rpm for 4 days at 25 °C, and the seed culture was then transferred to 1 L Erlenmeyer flasks containing 500 mL of A1+C liquid medium (total 12 L). The culture flasks were incubated aerobically with shaking at 200 rpm for 7 days at 25 °C. Secondary metabolites in the culture broth were analyzed daily from day 3 to 7 using an Agilent 1200 series LC/MS system equipped with a Phenomenex Luna C18(2) column (150 × 4.60 mm, 5 μm) and eluted with a linear aqueous acetonitrile gradient from 10 to 100% in 0.05% formic acid at a flow rate of 0.7 mL/min. The culture broth (day 7) of strain KME 002^T^ was extracted twice with ethyl acetate (1:1, v/v). The ethyl acetate solutions were then decanted, filtered and concentrated under reduced pressure to yield 650 mg of crude extracts. 

### 3.4. Separation and Purification of Two New Bile Acid Derivatives, Compounds ***1*** and ***2***

The culture extracts from strain KME 002^T^ were initially separated using C18 flash column chromatography by step gradient elution with acetonitrile/water (20:80, 40:60, 60:40, 80:20 and 100:0) to produce five fractions (fractions I-V). Fraction III (79.0 mg) was dried *in vacuo* and fractionated by preparative HPLC using an isocratic elution of 45% acetonitrile in water containing 0.02% trifluoroacetic acid for 30 min (flow rate 4 mL/min, Phenomenex Luna C18(2), 10 × 250 mm, 10 μm) to yield four subfractions (fractions III-1 to III-4). Compound **1** (7.9 mg, *t*_R_ 23.5 min) was purified from subfraction III-2 (10.5 mg) by preparative HPLC using isocratic elution with 35% acetonitrile in water containing 0.02% trifluoroacetic acid for 30 min (flow rate 4 mL/min, Phenomenex Luna C18(2), 10 × 250 mm, 10 μm). Fraction IV (36.9 mg) was dried *in vacuo* and fractionated by preparative HPLC using isocratic elution with 50% acetonitrile in water containing 0.02% trifluoroacetic acid for 1 h (flow rate 4 mL/min, Phenomenex Luna C18(2), 10 × 250 mm, 10 μm) to yield four subfractions (fractions IV-1 to IV-4). Compound **2** (3.2 mg, *t*_R_ 13.0 min) was purified from subfraction IV-4 (4.8 mg) by preparative HPLC using isocratic elution with 60% acetonitrile in water containing 0.02% trifluoroacetic acid for 30 min (flow rate 4 mL/min, Phenomenex Luna C18(2), 10 × 250 mm, 10 μm).

*3,3,12-Trihydroxy-7-ketocholanic acid* (**1**): colorless oil; 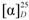: +19.0 (*c* 0.10, EtOH); IR (film) *v*_max _3479, 3056, 2952, 1714, 736 cm^−1^; ^1^H- and ^13^C-NMR spectra: see Table 1; HRFABMS [M–H_2_O+Na]^+^* m/z* 427.2451 (calcd. for C_24_H_36_O_5_Na, 427.2460). 

*3,3,12-Trihydroxy-7-deoxycholanic acid* (**2**): colorless oil; 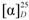: +25.0 (*c* 0.10, EtOH); IR (film) *v*_max_ 3444, 2939, 2868, 1700, 1206, 1140 cm^−1^; ^1^H- and ^13^C-NMR spectra: see Table 1; HRFABMS [M–H_2_O+Na]^+^*m/z* 413.2662 (calcd. for C_24_H_38_O_4_Na, 413.2668).

## 4. Conclusions

Two new cholic acid ketals were isolated from the new marine-derived bacterium *Hasllibacter halocynthiae *KME 002^T^. The basic structural features of both compounds **1** and **2**, identified by spectroscopic methods, including 2D-NMR experiments, was a 12-hydroxycholanic acid core with a unique 3,3-dihydroxy group. For decades, investigation at the chemical and biochemical levels to better understand the chemical diversity of microbial secondary metabolites has made continuous and impressive progress in the field of natural products. The isolation of **1** and **2** is expected to contribute to a better understanding of the structural diversity of cholic acid derivatives.
